# Monocyte-to-lymphocyte ratio: a potential novel predictor for acute kidney injury in the intensive care unit

**DOI:** 10.1080/0886022X.2022.2079521

**Published:** 2022-06-07

**Authors:** Fen Jiang, Jie Lei, Jiaxuan Xiang, Yuanhan Chen, Jingsheng Feng, Wenhe Xu, Jihong Ou, Bo Yang, Li Zhang

**Affiliations:** a Division of Nephrology, The First Affiliated Hospital of Hengyang Medical School, University of South China, Hengyang, China; b Division of Nephrology, Guangdong Provincial People's Hospital, Guangdong Academy of Medical Sciences, Guangzhou, China; c Division of Clinical Medicine, Hengyang Medical College of South China University, Hengyang, China

**Keywords:** Monocyte, lymphocyte, neutrophil, inflammation, acute kidney injury, intensive care unit

## Abstract

Monocyte-to-lymphocyte ratio (MLR) and neutrophil-to-lymphocyte ratio (NLR) are considered as surrogate inflammatory indexes. Previous studies indicated that NLR was associated with the development of septic acute kidney injury (AKI). The objective of the present study was to explore the value of MLR and NLR in the occurrence of AKI in intensive care unit (ICU) patients. The clinical details of adult patients (*n* = 1500) who were admitted to the ICU from January 2016 to December 2019 were retrospectively examined. AKI was diagnosed according to the Kidney Disease: Improving Global Outcomes criteria. The development of AKI was the main outcome, and the secondary outcome was in-hospital mortality. Overall, 615 (41%) patients were diagnosed with AKI. Both MLR and NLR were positively correlated with AKI incidence (*p* < 0.001). Multivariate logistic regression analysis suggested that the risk value of MLR for the occurrence of AKI was nearly three-fold higher than NLR (OR = 3.904, 95% CI: 1.623‒9.391 vs. OR = 1.161, 95% CI: 1.135‒1.187, *p* < 0.001). The areas under the receiver operating characteristic curve (AUC) for MLR and NLR in the prediction of AKI incidence were 0.899 (95% CI: 0.881‒0.917) and 0.780 (95% CI: 0.755‒0.804) (all *p* < 0.001), with cutoff values of 0.693 and 12.4. However, the AUC of MLR and NLR in the prediction of in-hospital mortality was 0.583 (95% CI: 0.546‒0.620, *p* < 0.001) and 0.564 (95% CI: 0.528‒0.601, *p* = 0.001). MLR, an inexpensive and widely available parameter, is a reliable biomarker in predicting the occurrence of AKI in ICU patients.

## 1. Introduction

Acute kidney injury (AKI) is a complicated clinical disorder and is frequently encountered in intensive care unit (ICU) [1,2]. Since AKI could result in permanent injuries to the kidney and long-term mortality [3,4], prediction and early intervention for AKI are of paramount importance.

Currently, AKI diagnosis is based on urine volume and serum creatinine (Scr) levels; however, these have limited significance in the early diagnosis of the condition [5]. Given the elevated AKI incidence in the ICU along with its dismal prognosis, a growing number of observational investigations have attempted to identify clinical indicators to estimate mortality in individuals developing AKI [6,7]. Several novel biomarkers, for instance neutrophil gelatinase-associated lipocalin (NGAL) along with Cystatin C (CystC), have been investigated for their role in early AKI detection [5,8,9]. However, they have not yet been widely applied in clinical practice. Therefore, identification of ideal biomarkers for AKI diagnosis is clinically important.

Inflammation is a central component in the onset and progress of AKI. Patients with AKI present with changes in morphology and function of vascular endothelial cells [10–12]. Monocyte-to-lymphocyte ratio (MLR) and neutrophil-to-lymphocyte ratio (NLR) are reliable inflammatory biomarkers that are calculated from complete blood counts [13,14]. Moreover, it has been reported that the NLR is tied to the onset and progress of AKI [15,16]. However, the role of MLR in the incidence and prognosis of AKI is still unclear. Therefore, the purpose of this study was to assess the diagnostic and prognostic significance of MLR and NLR in AKI estimation in critically ill patients.

## 2. Methods

### 2.1. Study design

This was a retrospective study. All patients admitted to the ICU at the First Affiliated Hospital of University of South China between 1 January 2016 and 31 December 2019 was considered for the study. Ethics approval of the study was given by the first affiliated hospital of University of South China (certification number 20201211LL012).

### 2.2. Selection criteria

Patients were excluded from our study if: (1) they had a diagnosis of chronic renal insufficiency, (2) or kidney transplantation, (3) were admitted to the ICU for <24 h, (4) were younger than 18 years, (5) had a second admission to the ICU, (6) underwent dialysis within one month prior to admission or at the time of being admitted to the ICU, and (7) had no renal function or blood routine test data within two days following admission to the ICU.

AKI diagnosis was based on Kidney Disease: Improving Global Outcomes categorization definition [17] and patients were subsequently stratified into two groups: a non-AKI and an AKI group. We divided the NLR into quartiles: ≦6.5; >6.5 and ≦10.33; >10.330 and ≦14.575; and >14.575. We also divided the MLR into quartiles: ≦0.4260; >0.4260 and ≦0.5856; >0.5856 and ≦0.843; and >0.843. The lowest value of creatinine detected in the emergency clinic or general ward before being admitted to the ICU was considered as the baseline creatinine value. When this value could not be procured, it was obtained by using the modification of diet in renal disease formula, by assuming that the normal glomerular filtration rate is 75 mL min^−1^·1.73 m^2^ [18].

### 2.3. Data extraction

Data on patient demographics (age and sex), complete blood count, blood biochemistry, inflammatory markers, renal function, and primary disease were documented for every patient. The baseline features utilized were those recorded within 24 h of ICU admission. Blood biochemistry consisted of albumin, triglyceride, Scr, and blood urea nitrogen (BUN). The Acute Physiology and Chronic Health Evaluation II (APACHE II) score was calculated at the time of admission to the ICU for all critically ill patients.

### 2.4. Statistical analysis

In case of data, normally distributed continuous variables were expressed as the mean and standard deviation (mean ± SD), while abnormally distributed continuous variables were shown as the median and interquartile range (median). Comparisons between continuous variables were made using Wilcoxon rank-sum and t-tests. The categorical variables were compared by the chi-squared test or the Fisher exact test. Spearman’s correlation was applied to analyze the association of MLR with other variables. We divided the MLR and NLR into quartiles and analyzed each quartile individually. Multi-logical regression was used to test associations between the MLR and NLR with AKI development and prognosis, and the results were presented as odd ratios (ORs). The receiver operating characteristic (ROC) curve was applied to assess the predictive value of MLR and NLR to the development of AKI and in-hospital mortality. Cutoff values along with sensitivity and specificity of variables were determined by the Youden index. Two tailed *p* < 0.05 signified statistical significance for all the analyses. Complete statistical analysis was conducted with SPSS 16 software (Chicago, IL, USA).

## 3. Results

### 3.1. Baseline characteristics

**Figure 1.**
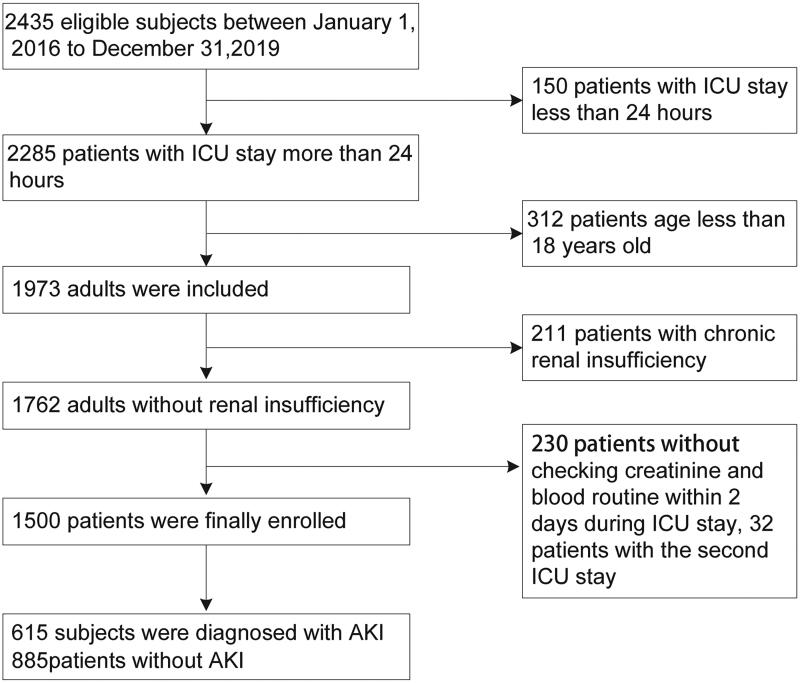
Flowchart of subject screening. ICU, intensive care unit; Scr, serum creatinine.

After reviewing the records of 2435 eligible participants who were admitted to the ICU at the First Affiliated Hospital of South China University between 1 January 2016 and 31 December 2019, 1500 patients were enrolled in the study (Figure 1).

Patients’ baseline characteristic and hematological data are shown in Table 1. The participants included 951 men and 549 women, with a mean age of 60.1 ± 16.14 years. The mean ± SD was 0.64 ± 0.31 for MLR and 12.29 ± 9.92 for NLR. Overall, 615 patients (41%) were grouped into the AKI group (151 patients in stage 1, 184 stage 2, and 272 in stage 3) and 885 patients (59%) were allocated to the non-AKI group.

**Table 1.** Baseline characteristics of the study population.

Variables	Total cohort (*n* = 1500)	Patients with AKI (*n* = 615)	Patients without AKI (*n* = 885)	*p-*value
Male (%)	951 (63.4%)	404 (42.5%)	547 (57.5%)	0.125
Age (years)	60.1 ± 16.14	63.27 ± 16.89	58.91 ± 16.73	<0.001*
Comorbidities				
Hypertension	486 (32.4%)	223 (36.3%)	263 (29.7%)	0.008*
Diabetes mellitus	225 (14.9%)	127 (20.7%)	96 (10.8%)	<0.001*
Coronary artery disease	263 (17.5%)	144 (23.4%)	119 (13.4%)	<0.001*
COPD	135 (9%)	48 (7.8%)	87 (9.8%)	0.178
Malignancies	74 (4.9%)	23 (3.7%)	51 (5.8%)	0.089
Severe liver disease	66 (4.4%)	41 (6.7%)	25 (2.8%)	0.003
Rheumatism	12 (0.8%)	5 (0.8%)	7 (0.8%)	0.590
Shock	147 (9.8%)	102 (16.6%)	45 (5.1%)	<0.001*
Hematologic disease	119 (7.9%)	65 (10.6%)	54 (6.1%)	<0.001*
Acute pancreatitis	81 (5.4%)	36 (5.9%)	45 (5.1%)	0.487
Acute infection	1021 (68.1%)	458 (74.5%)	563 (63.6%)	<0.001*
Sepsis	182 (12.1%)	127 (20.6%)	55 (6.2%)	<0.001*
Laboratory parameters				
Baseline Scr (μmol/L)	100.64 ± 9.8	127.34 ± 14.73	82.09 ± 19.25	<0.001*
BUN (mmol/L)	10.97 ± 9.25	16.58 ± 10.99	7.07 ± 4.91	<0.001*
Albumin (g/L)	33.45 ± 6.91	32.16 ± 4.79	34.35 ± 6.85	0.364
Triglyceride (mmol/L)	1.15 (0.79,1.72)	1.23 (0.87,1.24)	1.09 (0.75,1.66)	0.002*
PCT (ng/mL)	0.52 (0.17,3.88)	1.86 (0.31,16)	0.26 (0.14,1.4)	<0.001*
White blood count (10^9^/L)	11.77 (8.26,17.0)	12.23 (8.46,12.23)	11.59 (8.11,16.28)	0.786
Hemoglobin (g/L)	104.98 ± 29.75	103.06 ± 20.81	106.31 ± 29.65	0.038*
CystC (mg/L)	1.38 (0.89,2.72)	2.75 (1.86,4.6)	1.01 (0.75,1.41)	<0.001*
CRP (mg/L)	37.22 (8.28,95.24)	55.78 (17.02,131.59)	25.47 (4.6,76.08)	<0.001*
APACHEII	16.50 ± 7.32	19.09 ± 7.42	16.69 ± 6.7	<0.001*
MLR	0.64 ± 0.31	0.88 ± 0.29	0.47 ± 0.19	<0.001*
NLR	12.29 ± 9.92	17.02 ± 12.90	9.00 ± 5.03	<0.001*
PLR	188.16 ± 129.2	277.3 ± 133.5	153.1 ± 111.3	<0.001*

*p < 0.05.

AKI: acute kidney injury; APACHEII: acute physiology and chronic health evaluation II; BUN: blood urea nitrogen; COPD: chronic obstructive pulmonary disease; CRP: C-reactive protein; CystC: cystatin C; ICU: intensive care unit; MLR: monocyte-to-lymphocyte ratio; NLR: neutrophil-to-lymphocyte ratio; PCT: procalcitonin; PLR: platelet-to-lymphocyte ratio; Scr: serum creatinine.

The Scr, age, hemoglobin, BUN, Cyst C, C-reactive protein (CRP), procalcitonin (PCT), APACHE II scores, MLR, and NLR were remarkably higher in the AKI than the non-AKI group (*p* < 0.05). Remarkable differences indicated primary disease, specifically hypertension, coronary artery disease, severe liver disease, shock, acute infection, sepsis, hematologic disease, and diabetes mellitus in the two groups (*p* < 0.05). No significant difference in sex, albumin, white blood cells, and primary disease in terms of malignancies, rheumatism, acute pancreatitis and chronic obstructive pulmonary disease (*p* > 0.05) were observed in the groups.

### 3.2. MLR-related factors at baseline

Baseline MLR was positively linked to the CRP level, age, PCT, NLR and baseline Scr, and BUN (*p* < 0.001). Baseline MLR was negatively associated with the level of albumin with *r* = –0.142 (*p* < 0.001; Table 2).

**Table 2.** The relationship of baseline MLR with various factors.

Variables	*R*	*p-*value
Age (years)	0.094	<0.001*
Baseline Scr (μmol/L)	0.161	<0.001
BUN (mmol/L)	0.437	<0.001
Albumin (g/L)	–0.142	<0.001
Triglyceride (mmol/L)	0.042	0.539
CRP (mg/L)	0.148	<0.001
PCT (ng/mL)	0.243	<0.001
APACHE II	0.170	<0.001
NLR	0.344	<0.001*
PLR	0.387	<0.001*

*p < 0.05.

APACHE II: acute physiology and chronic health evaluation II; BUN: blood urea nitrogen; CRP: C-reactive protein; MLR: monocyte-to-lymphocyte ratio; NLR: neutrophil-to-lymphocyte ratio; PCT: procalcitonin; PLR: platelet-to-lymphocyte ratio; Scr: serum creatinine.

### 3.3. MLR predicts the incidence of AKI

A significantly higher baseline MLR and NLR was observed in AKI group in contrast with that of non-AKI group (*p* < 0.05). We divided MLR and NLR into four intervals according to the quartile interval, and the mean MLR and NLR values of the patients were used. Patients with a higher MLR quartile or NLR quartile had an elevated AKI incidence rate; quartile four had the highest AKI incidence rate (*p* < 0.001; Table 3).

**Table 3.** Comparison of baseline MLR and NLR between patients with AKI or non-AKI.

	Patients with AKI	Patients without AKI	*p*-value
MLR			
First quartile	32	342	<0.001*
Second quartile	44	330	<0.001*
Third quartile	194	181	0.502
Fourth quartile	343	32	<0.001*
NLR			
First quartile	300	70	<0.001*
Second quartile	273	100	<0.001*
Third quartile	228	147	0.002*
Fourth quartile	84	291	<0.001*

*p < 0.05.

AKI: acute kidney injury; MLR: monocyte-to-lymphocyte ratio; NLR: neutrophil-to-lymphocyte ratio.

Logistical regression was used to analyze the correlations of MLR and NLR with AKI during admission to the ICU, after adjusting for baseline Scr, age, sex, malignancies, severe liver disease, rheumatism, acute infection, shock, acute pancreatitis, hematologic disease, diabetes mellitus, coronary artery disease, hypertension, and chronic obstructive pulmonary disease. This analysis showed that MLR and NLR were tied to the incidence of AKI in individuals who were critically ill, with ORs of 3.904 and 1.161 (*p* < 0.001; Table 4). However, when MLR and NLR were combined, the ORs reduced to 1.377, suggesting that MLR was a better indicator of AKI incidence. The ORs of CRP, PCT, and APACHE II score were 1.004, 1.025, and 1.079 (*p* < 0.001).

**Table 4.** Values of the MLR and NLR for AKI by multivariate logical regression analysis.

Variables	Unadjusted		Adjusted	
	OR (95% CI)	*p*-value	OR (95% CI)	*p*-value
MLR	4.007 (1.772, 9.06)	<0.001	3.904 (1.623, 9.391)	<0.001*
NLR	1.161 (1.137, 1.186)	<0.001	1.161 (1.135, 1.187)	<0.001*
CRP (mg/L)	1.005 (1.004, 1.007)	<0.001	1.004 (1.003, 1.006)	<0.001*
PCT (ng/mL)	1.026 (1.020, 1.032)	<0.001	1.025 (1.019, 1.031)	<0.001*
CombindMLR + NLR	1.394 (1.344, 1.446)	<0.001	1.377 (1.325, 1.431)	<0.001*
Hemoglobin (g/L)	0.996 (0.993, 1.000)	0.038	0.996 (0.992, 1.000)	0.039*
Albumin (g/L)	0.954 (0.939, 0.969)	<0.001	0.953 (0.937, 0.970)	<0.001*
Triglyceride (mmol/L) (mmol/L) (mmol/L)	1.090 (1.028, 1.157)	0.004	1.087 (1.022, 1.156)	0.008*
APACHE II	1.092 (1.075, 1.109)	<0.001	1.079 (1.060, 1.097)	<0.001*

*Adjusted for age, sex, malignancies, severe liver disease, rheumatism, acute infection, shock, acute pancreatitis, hematologic disease, baseline Scr, diabetes mellitus, coronary artery disease, hypertension, and chronic obstructive pulmonary disease.

APACHEII: acute physiology and chronic health evaluation II; CI: confidence interval; CRP: C-reactive protein; MLR: monocyte-to-lymphocyte ratio; NLR: neutrophil-to-lymphocyte ratio; OR: odds ratio; PCT: procalcitonin; PLR: platelet-to-lymphocyte ratio.

### 3.4. Efficiency of the MLR for predicting AKI

**Figure 2.**
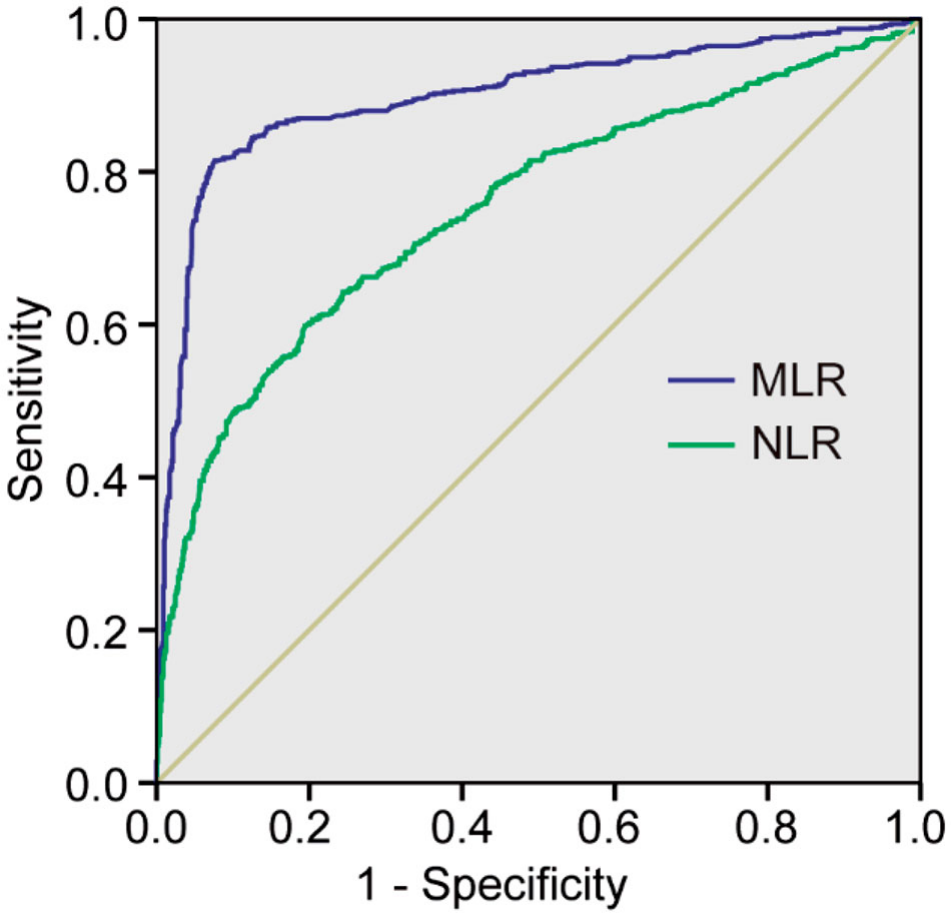
ROC curves for MLR and NLR for predicting AKI. MLR, monocyte-to-lymphocyte ratio; NLR, neutrophil-to-lymphocyte ratio.

ROC analysis was applied to examine the correlation of MLR and NLR for AKI incidence (Figure 2). The AUC of the MLR and NLR was 0.899 (95% CI: 0.881–0.917*, p* < 0.001) and 0.751 (95% CI: 0.726–0.777*, p* < 0.001), respectively. The best cutoff value of ROC was 0.693 for MLR with 81.5% sensitivity and 93.3% specificity. The cutoff value of ROC was 12.4 for NLR with 59.8% sensitivity and 80.6% specificity.

### 3.5. The relationship of MLR with in-hospital mortality

After adjusting for sex, age, malignancies, severe liver disease, rheumatism, acute pancreatitis, hematologic disease, baseline Scr and diabetes mellitus, coronary artery disease, hypertension, and chronic obstructive pulmonary disease, the ORs of MLR and NLR for in-hospital mortality were 1.716 (95% CI 1.122–2.625, *p* = 0.013) and 1.012 (95% CI 1.000–1.025, *p* = 0.048), respectively. Meanwhile, after combining MLR and NLR, the OR was 1.014 (95% CI 1.003–1.026, *p* = 0.019) for in-hospital mortality. The ORs of APACHEII, CystC and PCT for in-hospital mortality were 1.098 (1.007.1.119), 1.142 (1.067, 1.222) and 1.005 (1.001–1.009), respectively (both *p* < 0.05).AKI was positively associated with the in hospital mortality. However, acute infection, hemoglobin, and albumin did not show clear relationship with in hospital mortality (*p* > 0.05; Table 5).

**Table 5.** Univariable and multivariate logistic regression analysis of parameters to predict in-hospital mortality.

Variables	Unadjusted		Adjusted	
	OR (95% CI)	*p*-value	OR (95% CI)	*p*-value
MLR	2.321 (1.588, 3.456)	<0.001	1.716 (1.122, 2.625)	0.013
NLR	1.015 (1.003, 1.027)	0.011	1.012 (1.000, 1.025)	0.048
CRP (mg/L)	1.002 (1.000, 1.003)	0.078	1.001 (0.999, 1.003)	0.253
PCT (ng/mL)	1.006 (1.020, 1.010)	0.003	1.005 (1.001, 1.009)	0.019
Combined MLR + NLR	1.018 (1.007, 1.009)	0.001	1.014 (1.002, 1.026)	0.019
AKI	2.096 (1.618, 2.715)	<0.001	1.791 (1.354, 2.369)	<0.001
Hemoglobin (g/L)	0.997 (0.993, 1.001)	0.166	0.998 (0.993, 1.002)	0.338
Albumin (g/L)	0.983 (0.965, 1.002)	0.076	0.986 (0.967, 1.006)	0.178
APACHEII	1.103 (1.083, 1.123)	<0.001	1.098 (1.007, 1.119)	<0.001
Acute infection	1.381 (1.037, 1.389)	0.027	1.323 (0.984, 1.778)	0.064
CystC (mg/L)	1.183 (1.111, 1.260)	<0.001	1.142 (1.067, 1.222)	<0.001
Triglyceride (mmol/L)	1.014 (0.961, 1.069)	0.614	1.035 (0.980, 0.093)	0.217

*Adjusted for age, sex, malignancies, severe liver disease, rheumatism, acute pancreatitis, hematologic disease, baseline Scr, diabetes mellitus, coronary artery disease, hypertension, and chronic obstructive pulmonary disease.

AKI: acute kidney injury; APACHEII: acute physiology and chronic health evaluation II; CRP: C-reactive protein; MLR: monocyte-to-lymphocyte ratio; NLR: neutrophil-to-lymphocyte ratio; ORs: odds ratio; PCT: procalcitonin.

### 3.6. Ability of MLR for predicting in-hospital mortality

A total of 291 patients (19.4%) died in the hospital, of whom 162 (26.3%) patients were in the AKI group and 129 (14.6%) were in non-AKI group (data not shown) (*p* < 0.001). The ROC analysis was conducted to explore the prognostic values of MLR and NLR, which were plotted to analyze the relationship of predictors to in-hospital mortality individually. The AUC of the MLR and NLR for in-hospital mortality was 0.583 (95% CI: 0.546‒0.620, *p* < 0.001) and 0.564 (95% CI: 0.528‒0.601, *p* = 0.001), respectively. The best cutoff value for MLR was 0.687, with 51.2% sensitivity and 64.3% specificity; meanwhile, the best cutoff value for NLR was 11.77, with 48.5% sensitivity and 62.7% specificity (Table 6).

**Table 6.** ROC curve of the MLR and NLR for in-hospital mortality.

Variable	AUC (95% CI)	*p*-value	Cutoff values	Sensitivity (%)	Specificity (%)
MLR	0.583 (0.546‒0.620)	<0.001	0.687	51.2	64.3
NLR	0.564 (0.528‒0.601)	0.001	11.77	48.5	62.7

AUC: area under the curve; CI: confidence interval; MLR: monocyte-to-lymphocyte ratio; NLR: neutrophil-to-lymphocyte ratio; OR: odds ratio.

## 4. Discussion

Our study demonstrated that the initial MLR and NLR values at the time of ICU admission could be considered as independent risk factors for AKI incidence. The ability of MLR to predict AKI is superior to NLR. However, in terms of predicting the prognosis, MLR and NLR did not show clear advantages in predicting in-hospital mortality.

Monocytes are circulating leukocytes that are important for innate and adaptive immunity, inflammation, and tissue remodeling [19]. Neutrophils and lymphocytes are potential surrogate markers of inflammation [20]. In this study, our findings suggested that MLR is positively associated with CRP and PCT, thereby demonstrating that MLR could be an inflammatory marker.

A numbers of experimental and clinical studies have suggested that ischemia‒reperfusion and inflammation play critical roles in the pathophysiology of AKI [10–12]. Ischemia or reperfusion results in dysfunction of renal tubular epithelial cells, vascular endothelial cells, and leukocytes, which causes kidney immune homeostasis disorder, and the ensuing inflammation leads to the death of kidney parenchymal cell and finally results in AKI [21,22].

Parlar et al. confirmed that the NLR can predict AKI after coronary artery bypass graft procedures (*p* = 0.02), and showed that its predictive value is higher than that of CRP [23]. Similarly, Yang et al. conducted a prospective study in patients following cardiovascular surgery, and found that both pre-surgery and post-surgery neutrophil-to-lymphocyte platelet in AKI patients was higher than non-AKI patients (*p* < 0.001) [24]. A meta-analysis including 9766 hospitalized patients showed that NLR was an effective biomarker for the development of AKI [25]. Recently, a study including 222 sepsis patients also found that higher levels of NLR were associated with septic AKI [15]. However, in our study, the ability of MLR for predicting AKI is superior to NLR: the AUC for MLR and NLR was 0.899 and 0.75, respectively. As logistical regression was used to analyze the inflammatory markers of AKI, the ORs of MLR were 3.904-fold, and the ORs of NLR were 1.161-fold for AKI. Chen et al. investigated the application of the MLR in 53,939 physical examination cases and concluded that when MLR exceeds 0.3, the percentage of hypertensive individuals with renal injury was significantly increased [26]. The cutoff MLR value in our study was 0.693, which was higher than that in their study, perhaps because our study population involved critically ill patients.

We further examined the potential of MLR and NLR for predicting the prognosis. Previous studies have shown that inflammatory biomarkers including the MLR and NLR are important prognostic predictors in immune system diseases [13,14]. Huang et al. explored the correlation between the MLR and Guillain‒Barré syndrome in a cohort study, and showed a positive correlation when the cutoff MLR value was 0.235 [27]. Similarly, a retrospective study indicated that patients with an MLR value exceeding the cutoff of 0.36 had a remarkably increased risk of acute myocardial infarction for long-term cardiovascular events [28]. Lucca et al. conducted a study in clear cell renal cell carcinoma, and found that the predictive value of the MLR for survival is higher than that of the platelet-to-lymphocyte ratio (PLR) [21]. The meta-analysis conducted by Zhao *et a.l* suggested that NLR was associated with the all-cause mortality in patients with chronic kidney disease [29]. However, contrary to previous studies, MLR and NLR only shown weak predictability for in-hospital mortality, which could be because the study population comprised critically ill patients and patients with other complications; however, AKI was not the primary reason.

There are also limitations in the current study. First, as a single-center retrospective study with a population of a single ethnicity, it was a challenge to control bias and confounders. However, patients with fewer than 48 h of hospital stay and patients with fewer than two blood tests were excluded to avoid the selection bias. Therefore, the results of this study can still represent the epidemiological characteristics of AKI in the ICU. Second, we failed to compare the MLR and NLR with established novel renal injury biomarkers, for instance NGAL, kidney injury molecule-1, and CystC. Third, diagnosing AKI was on the basis of only increased Scr, whilst the role of urine output was ignored, since the frequent use of diuretics could affect the incidence of AKI. Finally, the study was limited to investigating the short-term prognosis, and did not analyze long-term prognosis; the latter will require longer-term follow-up of individuals with AKI.

## 5. Conclusions

Our study supports the independent estimation power of MLR and NLR for AKI incidence in critically ill patients. Moreover, the ability of MLR for predictive AKI is superior to NLR. MLR as an inexpensive and routinely reported indicator could provide valuable information in early diagnosis of patients with AKI.
